# Supplementary figures and images for: Genome-Wide Identification and Expression Profiles of C-Repeat Binding Factor Transcription Factors in Betula platyphylla under Abiotic Stress
Source: Int J Mol Sci. 2023 Jun 24;24(13):10573. doi: 10.3390/ijms241310573 (PMC10342014; doi:10.3390/ijms241310573)

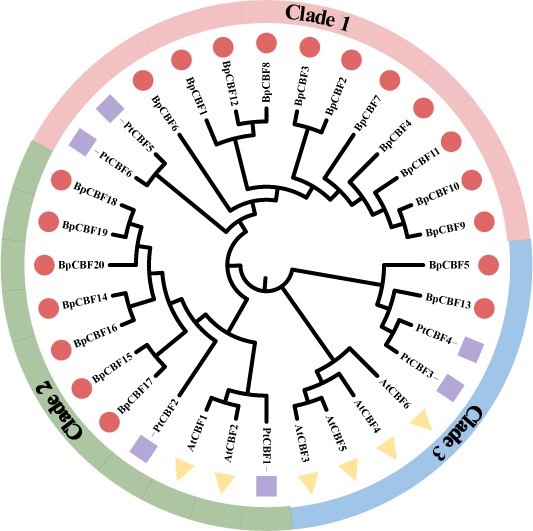

Supplement: Supplementary file 1 [file ijms-24-10573-s001.zip › Figure 1 Phylogenetic analysis of CBF proteins from Betula platyphylla, Arabidopsis tha-liana and Populus trichocarpa..jpg]

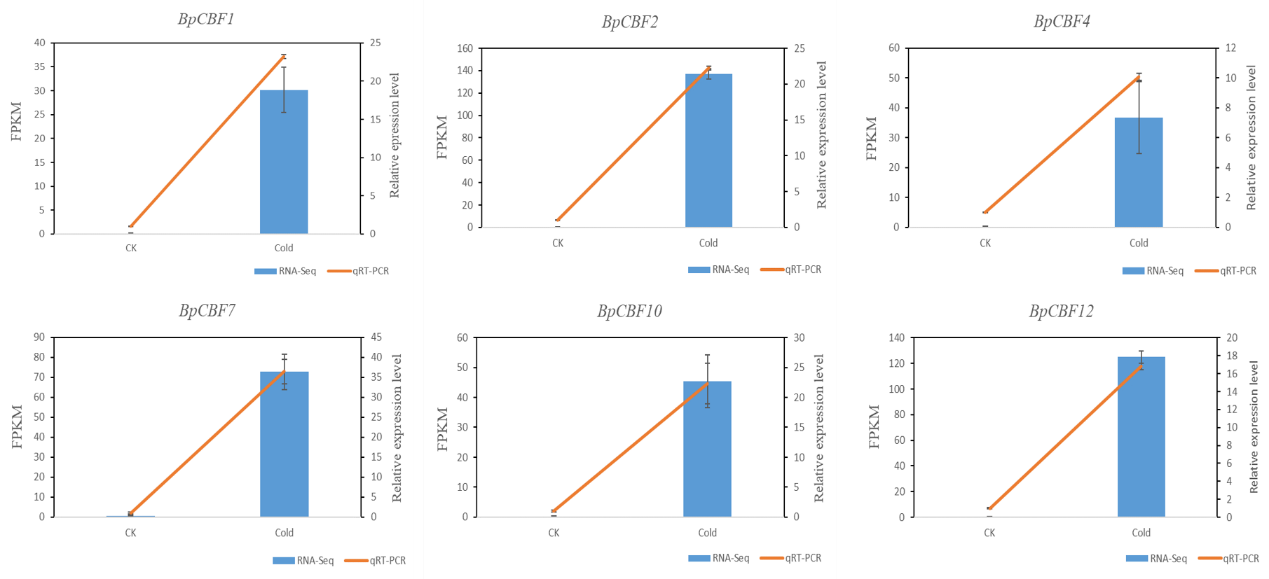

Supplement: Supplementary file 1 [file ijms-24-10573-s001.zip › Figure 10 The expression analysis of BpCBFs responding to cold stress by qRT-PCR and RNA-seq data under cold stress in Betula platyphylla leaves..png]

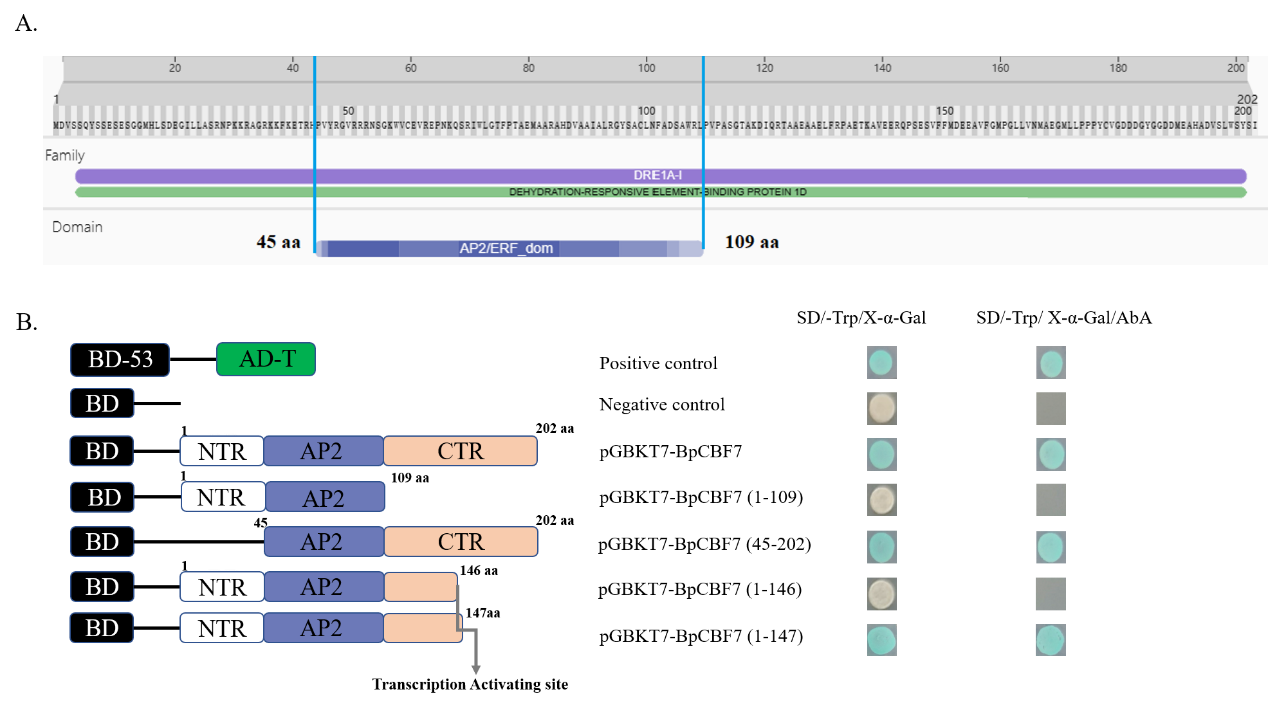

Supplement: Supplementary file 1 [file ijms-24-10573-s001.zip › Figure 11 Identification of transcription activating activity of BpCBF7..png]

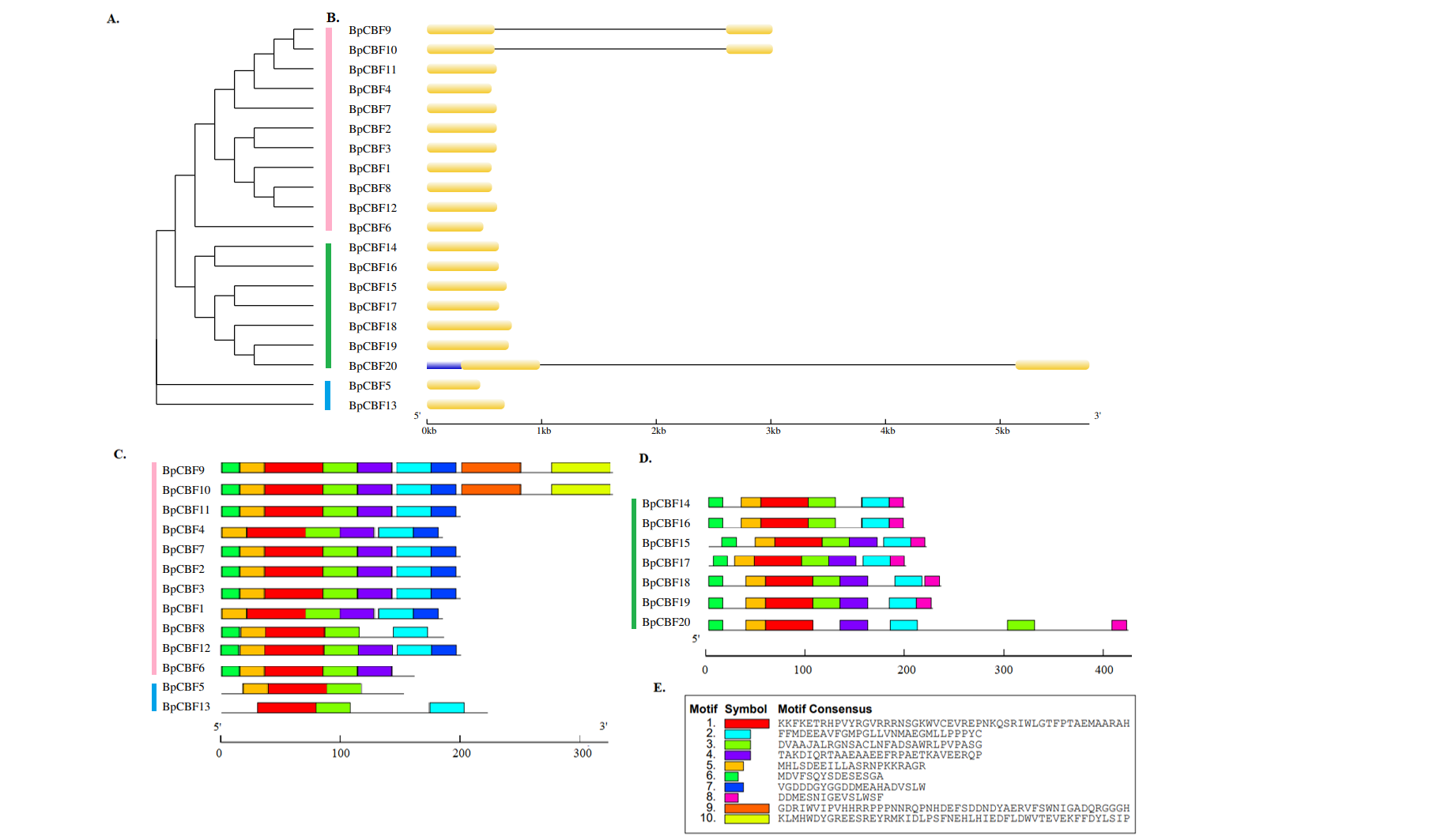

Supplement: Supplementary file 1 [file ijms-24-10573-s001.zip › Figure 2 Phylogenetic relationship, exon-intron gene structure, structure of conserved protein motifs in BpCBF transcription factors..png]

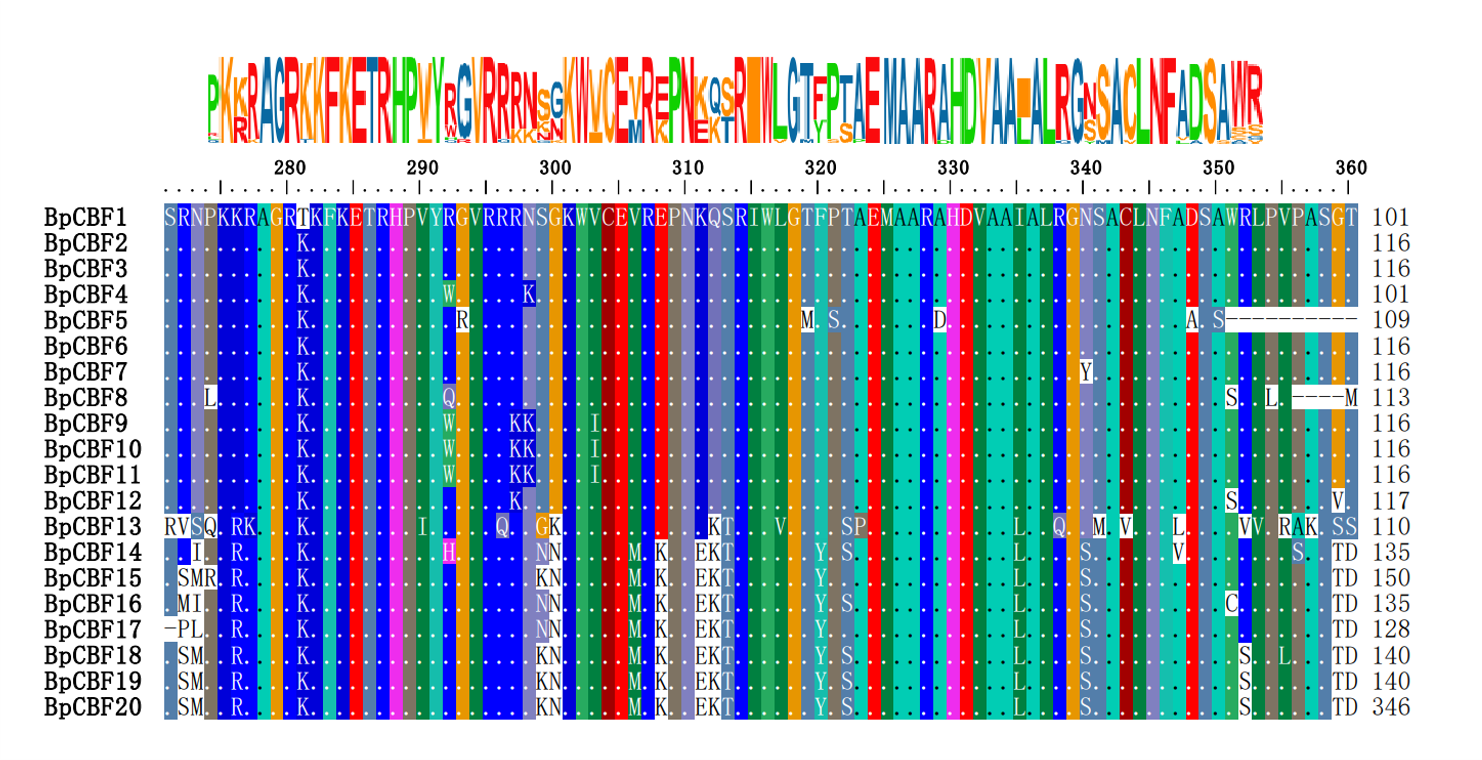

Supplement: Supplementary file 1 [file ijms-24-10573-s001.zip › Figure 3 Partial multiple sequence alignment analysis of the 20 BpCBFs..png]

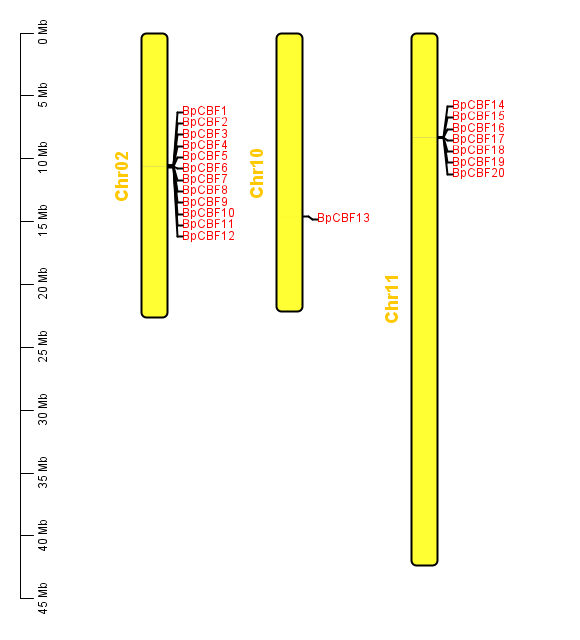

Supplement: Supplementary file 1 [file ijms-24-10573-s001.zip › Figure 4 Chromosome location of the BpCBFs..png]

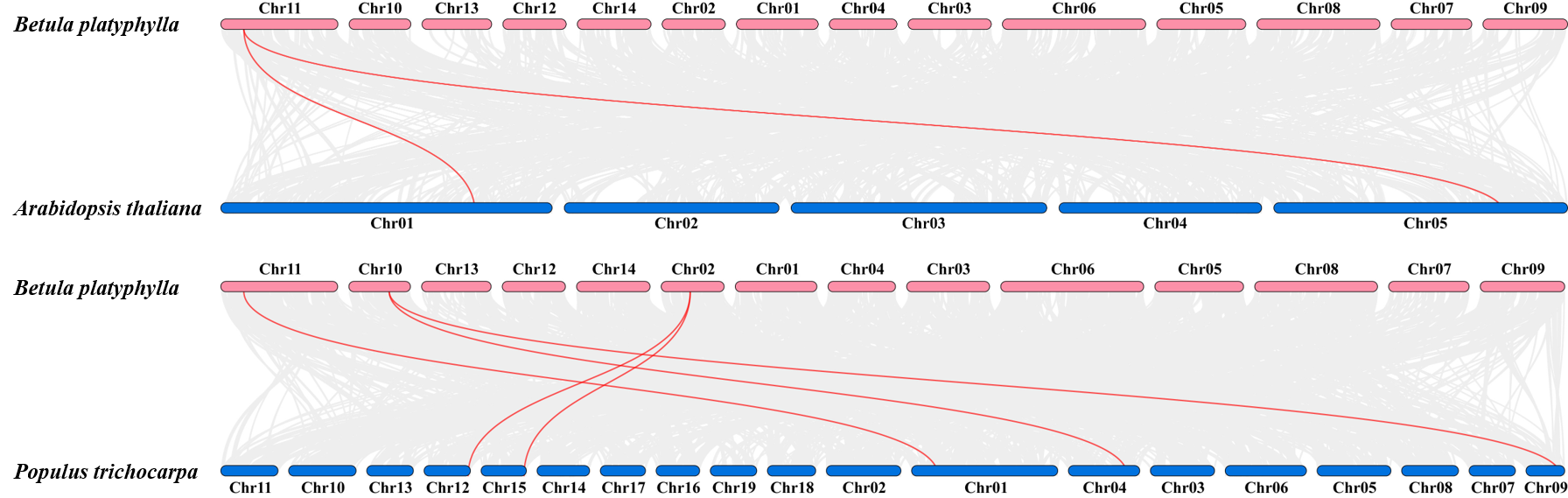

Supplement: Supplementary file 1 [file ijms-24-10573-s001.zip › Figure 5 Collinearity analysis of CBFs between Betula platyphylla and Arabidopsis thaliana, Betula platyphylla and Populus trichocarpa..png]

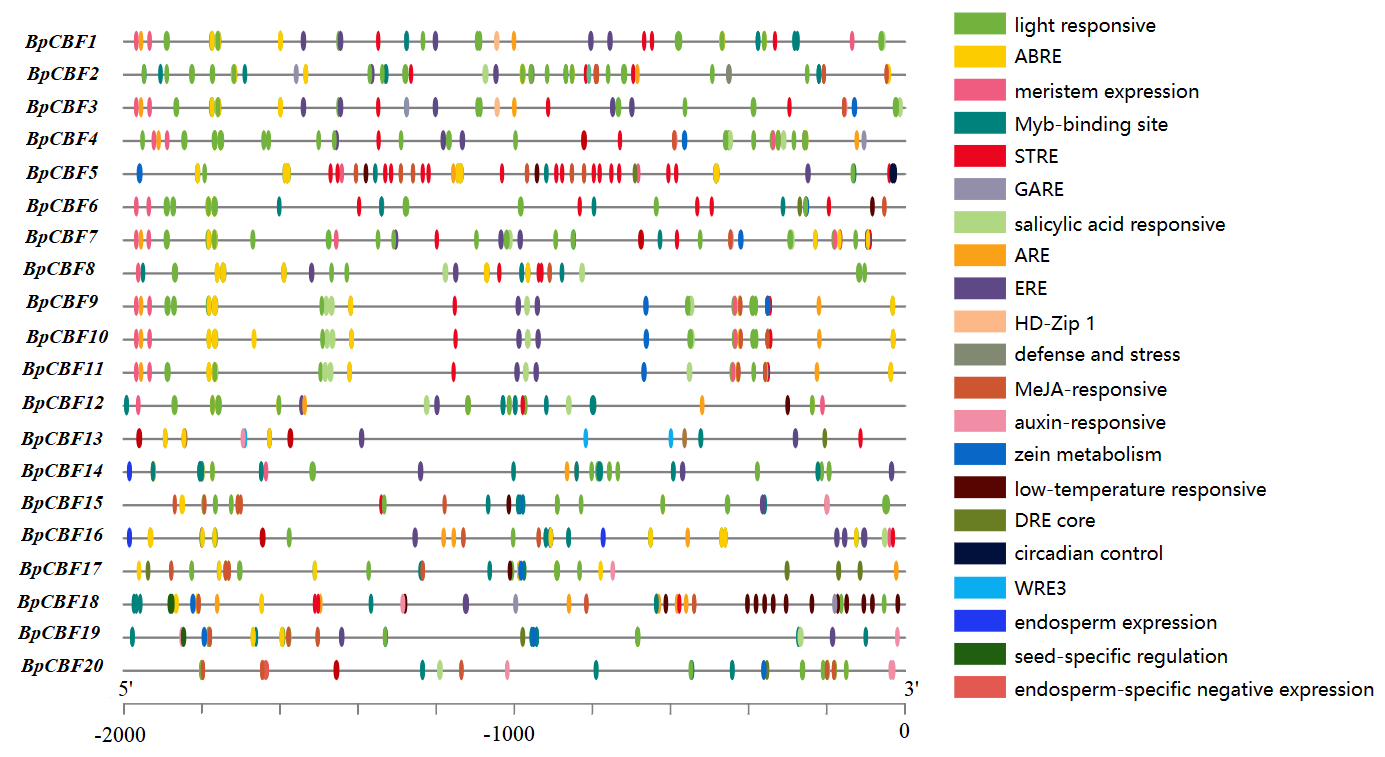

Supplement: Supplementary file 1 [file ijms-24-10573-s001.zip › Figure 6 Analysis of cis-acting elements in the promoters of the 20 BpCBFs..png]

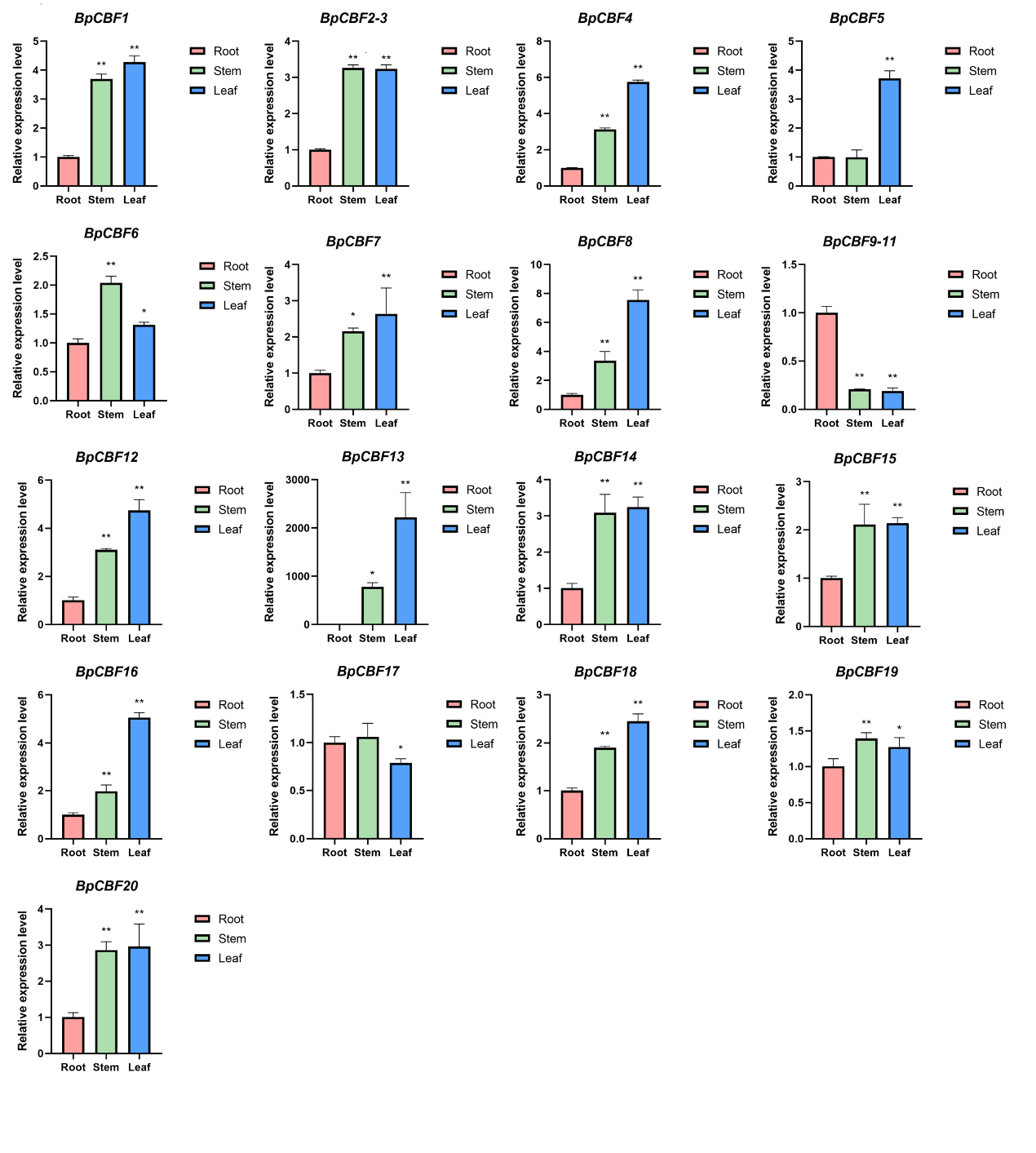

Supplement: Supplementary file 1 [file ijms-24-10573-s001.zip › Figure 7 Tissue-specific expression analysis of BpCBFs.png]

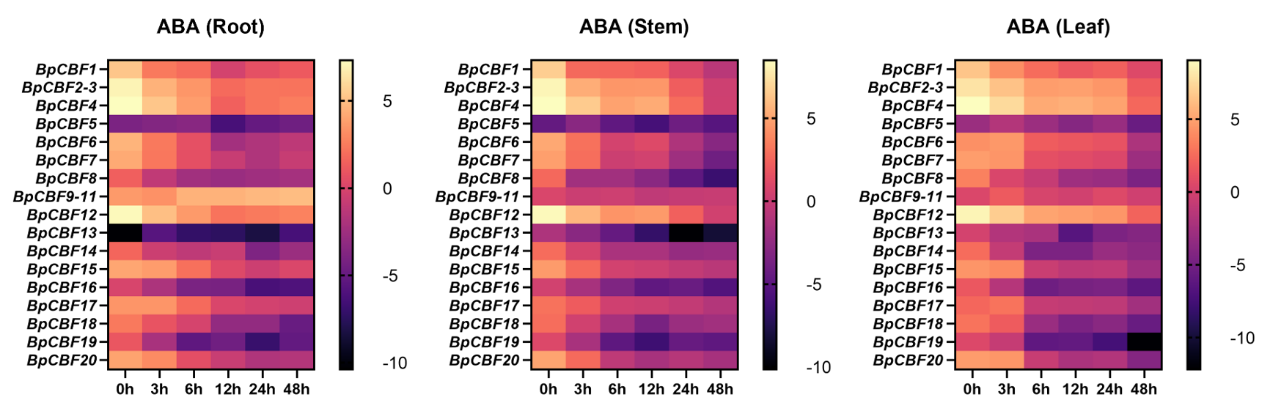

Supplement: Supplementary file 1 [file ijms-24-10573-s001.zip › Figure 8 Tissue-specific expression analysis of BpCBFs under ABA treatment by qRT-PCR..png]

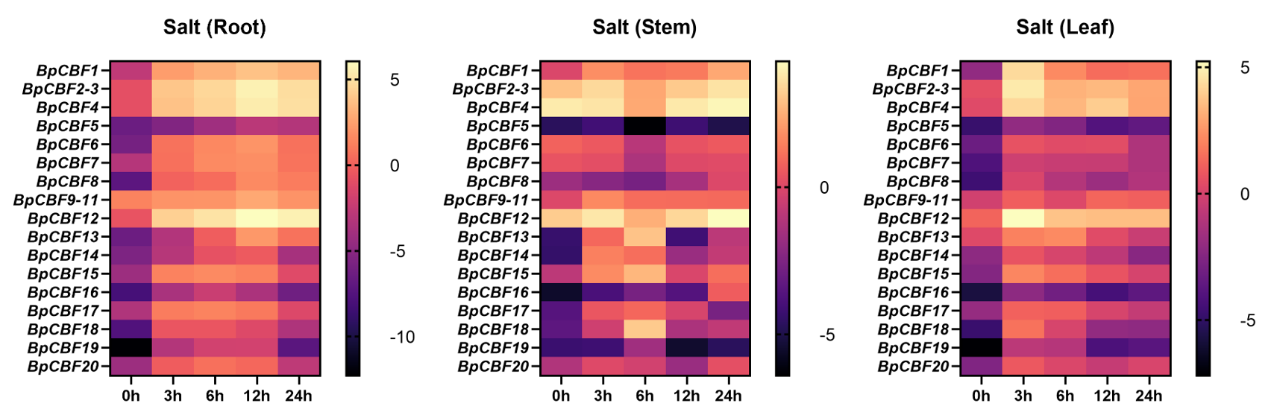

Supplement: Supplementary file 1 [file ijms-24-10573-s001.zip › Figure 9 Tissue-specific expression analysis of BpCBFs under salt stress by qRT-PCR..png]

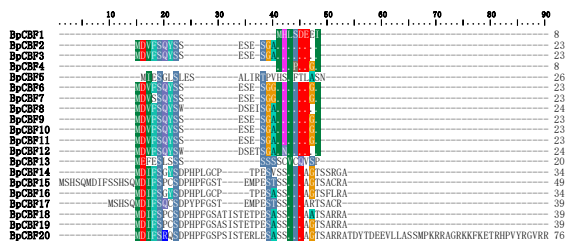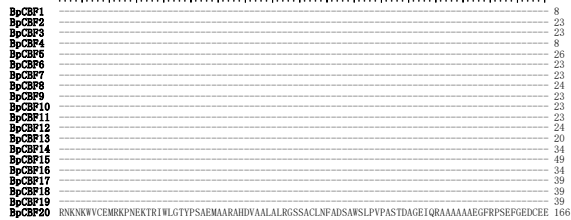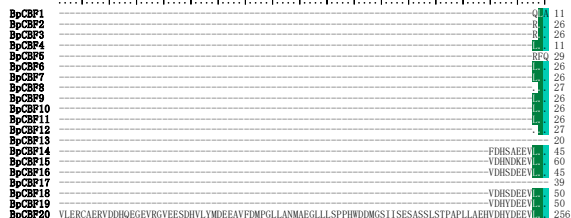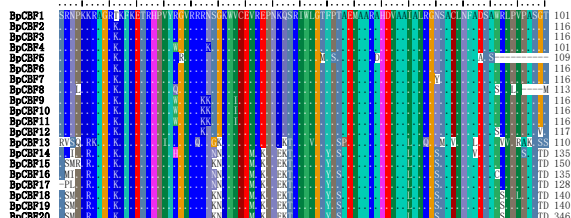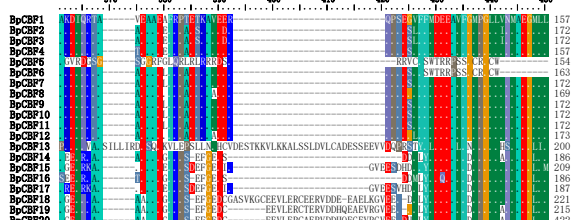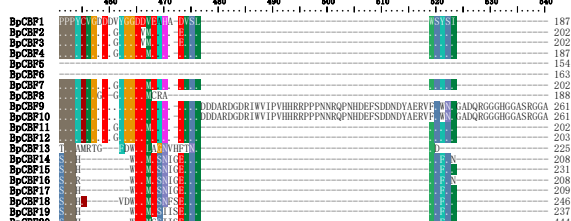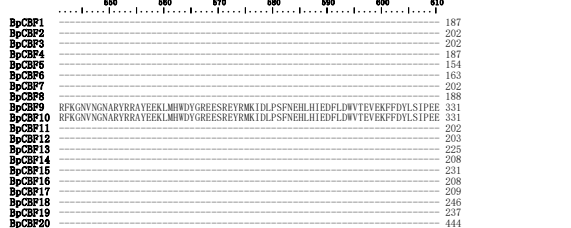

Supplement: Supplementary file 1 [file ijms-24-10573-s001.zip › Figure S1. Multiple sequence alignment analysis of 20 BpCBFs.pdf]
